# Development of a Curcumin-Loaded Hyaluronic Acid Nanogel Formulation Using Wet Granulation Method for Enhanced Dissolution and Stability

**DOI:** 10.3390/gels11080585

**Published:** 2025-07-29

**Authors:** Natkhanang Mookkie Boonpetcharat, May Thu Thu Kyaw, Veerakiet Boonkanokwong, Jittima Amie Luckanagul

**Affiliations:** 1 Department of Pharmaceutics and Industrial Pharmacy, Faculty of Pharmaceutical Sciences, Chulalongkorn University, Phyathai Road, Bangkok 10330, Thailand; natkhanang.b@gmail.com (N.M.B.); maythuhan117@gmail.com (M.T.T.K.); veerakiet.b@pharm.chula.ac.th (V.B.); 2 Center of Excellence in Plant-Produced Pharmaceuticals, Chulalongkorn University, Phyathai Road, Bangkok 10330, Thailand

**Keywords:** hyaluronic acid derivative, curcumin, delivery system, oral administration

## Abstract

Curcumin is widely recognized for its various pharmacological properties, including antioxidant, anti-inflammatory, and anti-tumor activities. Nevertheless, the development of curcumin as a therapeutic agent is impeded by its limited oral bioavailability, which stems from its chemical instability, poor aqueous solubility, and rapid degradation. This study aimed to develop granule formulations incorporating poly(N-isopropylacrylamide)-grafted hyaluronic acid or HA-g-pNIPAM to enhance dissolution and protect curcumin from degradation. Three formulations were developed: F10 (HA-g-pNIPAM physically mixed with curcumin), F10 Encap (curcumin encapsulated within HA-g-pNIPAM), and F11 (curcumin granules without HA-g-pNIPAM). The stability results showed that F10 Encap effectively maintained curcumin content throughout the study period, retaining approximately 94% of its initial concentration by day 30, compared to 70% from F11 (*p* < 0.05) at 30 °C and 75% relative humidity. All dried curcumin granules exhibited excellent flowability, as determined by the angle of repose measurements. All three formulations exhibited a consistent particle size distribution across replicates, with a peak in the 150–180 μm size range. The sustained release observed for F10 Encap and F10 after the initial burst suggested that the HA-g-pNIPAM provided a controlled release mechanism, ensuring continuous curcumin dissolution over 240 min in gastric and intestinal conditions. These findings suggested that HA-g-pNIPAM improved dissolution and stability of curcumin.

## 1. Introduction

Curcumin (C_21_H_20_O_6_) [1], a bioactive compound extracted from turmeric (*Curcuma longa*), has been widely recognized for its antioxidant, anti-inflammatory, antimicrobial, and potential anticancer properties. Its diverse pharmacological activities have contributed to its various therapeutic uses and dietary supplements. However, the development of curcumin as a therapeutic agent is hindered by its poor aqueous solubility, limited oral bioavailability, and rapid metabolism. Additionally, curcumin is prone to degradation during processing and in the gastrointestinal tract. Research indicates that these limitations can be overcome by nanotechnology [2].

Curcumin’s poor aqueous solubility and rapid metabolism have prompted extensive research into strategies to enhance its dissolution and bioavailability. Techniques such as solid dispersions, cyclodextrin complexation, lipid-based nanoemulsions, and polymeric nanoparticles have been explored to improve curcumin’s solubility. For instance, cyclodextrin complexes increased curcumin solubility for the treatment of inflammatory bowel disease (IBD) in colitis-induced rat model [3], while poloxamer-based solid dispersions demonstrated sustained release and enhanced solubility by disrupting curcumin’s crystalline lattice [4]. Moreover, nanoemulsions were proposed as a promising transdermal delivery system for curcumin, combining improved solubility, sustained release, and enhanced therapeutic efficacy for acne and wound healing applications [5].

Nanogels represent three-dimensional polymeric networks with crosslinked structures at the nanometer scale (generally ranging from 20 to 200 nm). These systems demonstrate exceptional water absorption capacity, excellent biocompatibility, and responsive behavior to environmental stimuli. Their adaptable architecture allows for effective incorporation of water-insoluble therapeutic agents, safeguarding them from breakdown while simultaneously improving their dissolution properties [6]. Nanogels stand out from other nanocarriers due to their high-water retention, flexibility, and tunable structure, making them uniquely effective for encapsulating hydrophobic bioactive compounds. Unlike rigid nanoparticles or lipid-based carriers (e.g., liposomes, nanoemulsions), nanogels offer a hydrophilic, three-dimensional network that provides superior biocompatibility, biodegradability, and controlled release properties. Their ability to swell and shrink in response to environmental conditions enables precise delivery and sustained release of encapsulated compounds [7]. Previous studies from our research group have established the potential of hyaluronic acid graft poly(N-isopropylacrylamide) (HA-g-pNIPAM) nanogels as an effective drug delivery system.

HA-g-pNIPAM nanogels combine the biocompatibility of hyaluronic acid with the thermoresponsive properties of pNIPAM, forming stable 100–300 nm particles. These nanogels exhibit temperature-dependent swelling, enzymatic biodegradability, and CD44-targeting capability. Their amphiphilic nature enables high drug loading and controlled pH/thermal-responsive release, while maintaining excellent colloidal stability (PDI < 0.3) and cytocompatibility, making them ideal for targeted drug delivery applications. One study investigated different media (ultrapure water, citrate buffer, and phosphate-buffer saline solution) to determine the most suitable conditions for self-assembly. It concluded that 0.5% *w*/*v* HA-g-pNIPAM in ultrapure water produces nanogels with a more desirable size and stability. This concentration was assessed for curcumin encapsulation, demonstrating enhanced solubility and stability compared to individual polymer components or nonmodified hyaluronic acid [8]. Another study further highlighted HA-g-pNIPAM as a biocompatible platform that significantly enhanced curcumin’s aqueous solubility, drug loading capacity, and biological activity, leading to improved cellular uptake in NIH-3T3 cells and greater therapeutic potential [9].

Building on these findings, the present study advances this nanogel formulation to make it more suitable for solid dosage forms, such as tablets, broadening its application in pharmaceutical development. This study transitions from fundamental nanogel characterization to addressing the practical formulation challenges of incorporating the HA-g-pNIPAM nanogel into a scalable oral solid dosage form via wet granulation, a complex process often encountered in the development of nanomedicines into pharmaceutically viable products [10]. Our unique contribution lies in systematically investigating the integration of this sophisticated nanogel into a conventional manufacturing process, comparing different nanogel incorporation strategies (physical mix vs. encapsulated) within a granule formulation, and evaluating their impact on dissolution and long-term stability under simulated gastrointestinal conditions.

Therefore, this study aims to compare three formulations: (1) F10 (HA-g-pNIPAM physically mixed with curcumin), (2) F10 Encap (curcumin encapsulated within HA-g-pNIPAM), and (3) F11 (curcumin granules without HA-g-pNIPAM). The findings will provide insights into the role of HA-g-pNIPAM nanogels in enhancing drug solubility, stability, and release properties, guiding future formulation optimization.

## 2. Results and Discussion

### 2.1. Stability of Curcumin in the Formulations

**Figure 1:**
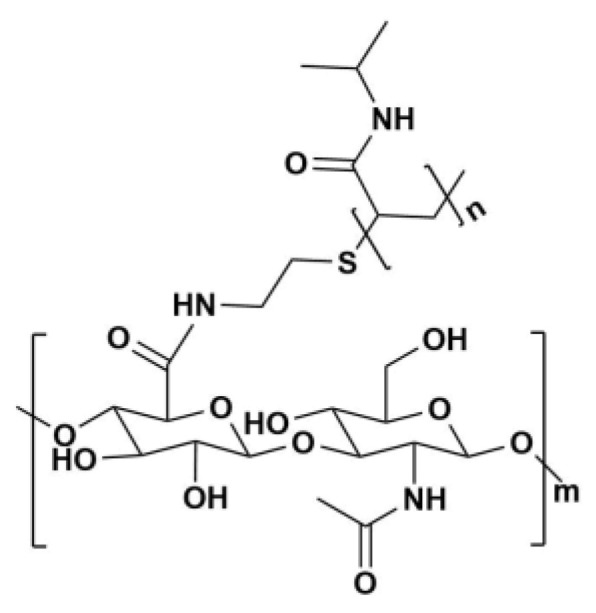
Chemical structure of HA-g-pNIPAM.

**Figure 2:**
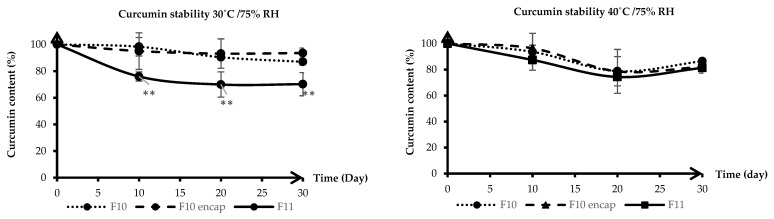
Stability of curcumin granules at 30 °C/75% RH (**left**) and 40 °C/75% RH (**right**) over 30 days. Lines represent mean curcumin content (%), and error bars indicate standard deviation (SD). F10 stands for HA-g-pNIPAM physically mixed with curcumin, F10 Encap symbolizes curcumin encapsulated within HA-g-pNIPAM, and F11 represents curcumin granules without HA-g-pNIPAM. ** indicates statistical significance at *p* < 0.05 (*n* = 3).

HA-g-pNIPAM (see Figure 1) was used in F10 and F10 Encap formulations. The content determination was performed following the United States Pharmacopeia (USP) method [11]. From Figure 2, on day 30 at 30 °C/75%RH, curcumin content in F11 dropped to about 70% which was significantly different from F10 (87%) and F10 Encap (94%) (*p* < 0.05). This suggested that the presence of HA-g-pNIPAM in the formulations contributed to the stability of curcumin, possibly by providing a protective environment that reduced degradation. Since curcumin is known to be sensitive to environmental factors such as light, heat, and oxygen, the lack of encapsulation might expose it more readily to these degradation pathways.

The results indicated that the stability of curcumin in all three formulations was affected by temperature and humidity. The increased temperature to 40 °C and the presence of 75% RH accelerated the degradation of curcumin, particularly in F11 granules, which lacked the protective HA-g-pNIPAM encapsulation. However, the encapsulated formulation demonstrated greater resistance to degradation at both temperatures, with F10 Encap showing slightly better stability than F10 at 40 °C. This could be due to the more effective encapsulation of curcumin within the HA-g-pNIPAM network, which might offer additional protection against the harsher conditions at 40 °C. However, further studies could explore the mechanisms of curcumin degradation in these formulations and the potential for further improvements in stability through formulation optimization or the addition of stabilizing agents.

### 2.2. Characterization of Dried Curcumin Granules

#### 2.2.1. Characteristics

According to Thai Herbal Pharmacopoeia, turmeric dry extract should not have more than 7% w/w of the initial weight after drying at 105 °C for 2 h [12]. The moisture content reported in Table 1, measured from granules of all formulations (F10, F10 Encap, and F11), demonstrated compliance with the specified criteria. Carr’s Index or compressibility index was determined using the bulk and tapped density values, which served as a measure of the compressibility of the granules. Granules are considered to have good compressibility if the Carr’s Index is below 15%, and fair compressibility if the index is below 20% [13]. The Carr’s Index reference table is provided in Supplementary Table S1. As shown in Table 1, F10 and F10 Encap exhibited fair compressibility, while F11 demonstrated good compressibility.

Another criterion to measure flowability was the angle of repose. If the angle of repose was less than 25 degrees, it indicated excellent flowability for a granular substance [13]. The angle of repose reference table can be found in Supplementary Table S2. The angle of repose refers to the maximum angle of inclination, in relation to the horizontal, at which a material can be stacked without collapsing. F10 demonstrated an average angle of repose of 9.5°, while F10 Encap showed 10° for all three replicates. F11 revealed an estimated 10.33°. At these values, all three formulations exhibited excellent powder flowability.

#### 2.2.2. Morphology

**Figure 3:**
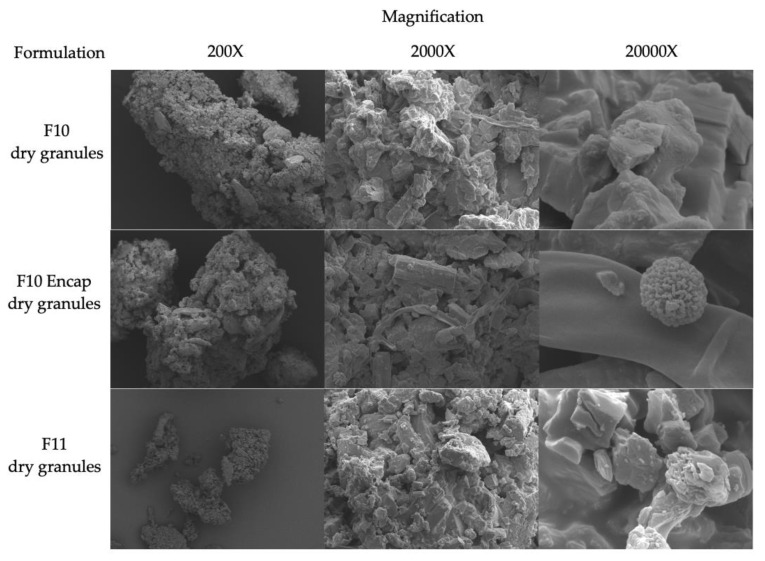
SEM images of F10 (HA-g-pNIPAM physically mixed with curcumin), F10 Encap (curcumin encapsulated within HA-g-pNIPAM), and F11 (curcumin granules without HA-g-pNIPAM) dried granules with magnification of 200×, 2000×, and 20,000×.

The scanning electron microscopy (SEM) images represented the surface texture and morphology of the granules (See Figure 3). The ingredients of base granules were curcumin, polyvinylpyrrolidone K-30 (PVP K-30), mannitol, and low-substituted hydroxypropyl cellulose (L-HPC). From previous studies, the shape of ingredients in granules appeared to coordinate with results from SEM images [14,15,16,17]. The image showed a rough surface; therefore, there is a gap for future studies to develop granules that contain smoother surfaces.

F10 and F10 Encap exhibited similar granule’s surface. F11 also showed the base granules’ surface which comprised curcumin, PVP K-30, mannitol, and L-HPC. The image complied with the previous studies [14,15,16,17].

#### 2.2.3. Particle Size Distribution

**Figure 4:**
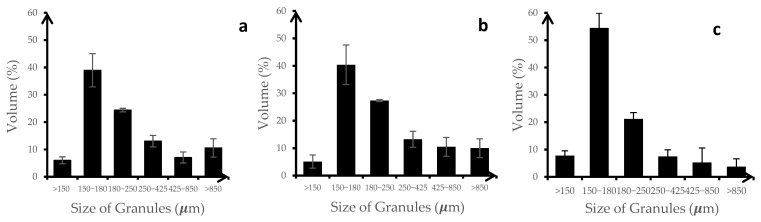
Particle size distribution of (**a**) F10 (HA-g-pNIPAM physically mixed with curcumin), (**b**) F10 Encap (curcumin encapsulated within HA-g-pNIPAM), and (**c**) F11 (curcumin granules without HA-g-pNIPAM), (*n* = 3).

The particle size distribution of the three formulations was relatively consistent across the three replicates, indicating good reproducibility in the granulation process (See Figure 4). The 150–180 μm size range was the most common size for the granules produced, which would be desirable for uniformity in drug delivery and processing. Shekunov et al. suggested that smaller particle sizes (20–50 μm) were optimal for chewable and fast-disintegrating tablets, while larger sizes (100–200 μm) were suitable for direct-compression tablets mostly because of their required compaction behavior and powder flow properties [18]. This suggested that all formulations showed suitable and consistent particle size distribution.

### 2.3. Characterization of Curcumin Reconstituted Granules

#### 2.3.1. Dissolution Test

**Figure 5:**
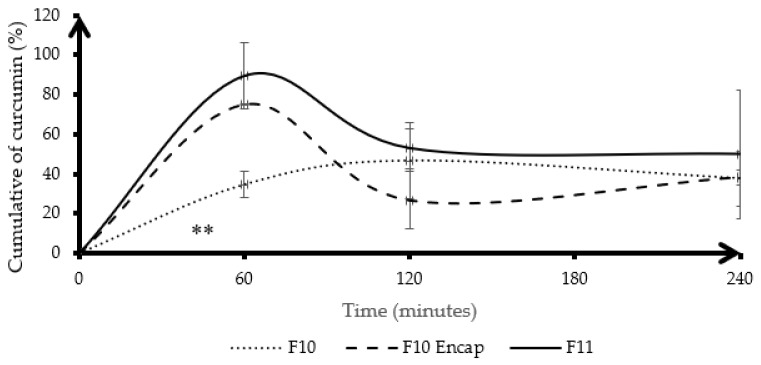
Cumulative dissolution of curcumin (%) in gastric condition. Lines represent the mean, and error bars indicate standard deviation (SD). F10 stands for HA-g-pNIPAM physically mixed with curcumin, F10 Encap symbolizes curcumin encapsulated within HA-g-pNIPAM, and F11 represents curcumin granules without HA-g-pNIPAM (*n* = 3). ** indicates statistical significance at *p* < 0.05.

Figure 5 illustrates the cumulative dissolution of curcumin over time under gastric conditions for three different formulations: F10 (curcumin physically mixed with HA-g-pNIPAM), F10 Encap (curcumin encapsulated within HA-g-pNIPAM), and F11 (curcumin granules without HA-g-pNIPAM). Among the three formulations, F11 showed the fastest and highest initial release, reaching a peak of approximately 90% at 60 min, likely due to the absence of a polymeric matrix, which allows rapid dissolution and diffusion of curcumin into the medium. In contrast, F10 Encap showed a moderately high release (around 75% at 60 min), indicating partial burst release from the encapsulated system. F10 Encap likely contained entrapped curcumin within its nanogel structure, differentiating it from F11. Consequently, the observed curcumin released from F10 Encap was more indicative of curcumin within the matrix rather than from the nanogel components. F10, which involved curcumin physically mixed with HA-g-pNIPAM, showed the slowest and most sustained release profile, with approximately 40% released at 60 min (*p* < 0.05) and a gradual increase over time. This behavior was different from F10 Encap and F11. The observed drug release profile reported in mass of curcumin measured by RP-HPLC, characterized by an initial major release around 60 min, followed by a subsequent decrease, and then a slight increase again, can be scientifically justified primarily by the instability of curcumin in highly acidic environments combined with the distinct release kinetics from our formulations. Curcumin is well-documented to degrade significantly over time in highly acidic conditions (pH 1.2) [19].

The dissolution profile of F10, without encapsulation, showed slower drug release compared to F10 Encap. However, more studies need to be conducted to ensure the encapsulation when formulated by directly adding nanogels without a prior encapsulation process. The interaction between curcumin and polymers could hinder the release of curcumin. Curcumin was bound with mannitol and L-HPC via a hydrogen bond, stronger than the interaction between curcumin and water in the formulation [20,21]. This suggested that while HA-g-pNIPAM alone may not improve dissolution when physically mixed, its encapsulation of curcumin could significantly enhance release compared to the unencapsulated form. The presence of other excipients—PVP K-30, mannitol, and L-HPC—likely contributes to the overall granule integrity and wetting properties but is not the main driver of dissolution differences observed among the three formulations.

F11 did not contain HA-g-pNIPAM, which could mean that its structure lacks a barrier or matrix that controls drug release, leading to a more rapid dissolution of the drug.

**Figure 6:**
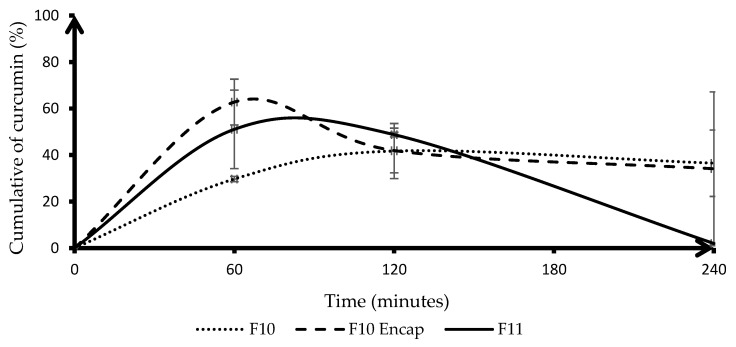
Cumulative dissolution of curcumin (%) in intestinal condition. Lines represent the mean, and error bars indicate standard deviation (SD). F10 stands for HA-g-pNIPAM physically mixed with curcumin, F10 Encap symbolizes curcumin encapsulated within HA-g-pNIPAM, and F11 represents curcumin granules without HA-g-pNIPAM, (*n* = 3).

The cumulative drug dissolution was monitored over 240 min in simulated intestinal conditions. Figure 6 presents the cumulative dissolution profiles of curcumin in simulated intestinal conditions for three formulations. F11 revealed notable differences in curcumin release behavior influenced by both formulation strategy and polymeric protection. F11, which contained curcumin granules without HA-g-pNIPAM, exhibited a rapid initial release, reaching approximately 55% at 60 min. However, this was followed by a marked decline, dropping to nearly 0% at 240 min. This sharp decrease suggested that curcumin, when unprotected, was highly unstable in intestinal environments, likely due to chemical degradation or precipitation at higher pH. In contrast, F10 Encap, where curcumin was encapsulated within HA-g-pNIPAM, showed a significantly higher initial release—peaking around 65%—which could be attributed to the burst release of surface-bound or loosely entrapped curcumin. F10, which consists of a physical mixture of curcumin and HA-g-pNIPAM, demonstrated the most sustained and stable release profile. It reached a moderate cumulative release of around 43% by 120 min. F10 and F10 Encap maintained dissolution level with minimal decline through 240 min. Compared to the dissolution profile under gastric conditions, F11 displayed different behavior. This suggested that the hydrogel network of HA-g-pNIPAM might protect curcumin from rapid environmental degradation and modulate its diffusion rate, making it more suitable for sustained release applications. Overall, these results underscore the importance of both polymer incorporation and formulation design in enhancing curcumin stability and release in the intestinal environment, with HA-g-pNIPAM providing notable protection against pH-induced degradation. The inclusion of excipients such as PVP K-30 (a binder), mannitol (a filler with osmotic activity), and L-HPC (a disintegrant) likely influenced the disintegration and wettability of the granules across all formulations, but the presence and type of HA-g-pNIPAM interaction with curcumin played a more significant role in modulating dissolution behavior.

Upon contact with the aqueous dissolution media, our nanogel-containing granules undergo rapid hydration and swelling, a fundamental characteristic of hydrogel-based systems that facilitates significant water uptake [22]. This initial swelling promotes granule disintegration and the subsequent relaxation of the nanogel’s polymer network, which in turn increases the mesh size and creates channels conducive to enhanced diffusion of the encapsulated curcumin out of the matrix [23]. Furthermore, our dissolution studies rigorously maintained sink conditions, ensuring that the large volume of the dissolution medium kept the concentration of released curcumin well below its saturation solubility. This continuous concentration gradient, enforced by the sink condition, served as a primary driving force for drug release, promoting rapid solubilization and dispersion of curcumin once it diffused from the swelling nanogel and granule matrix [24]. Therefore, the inherent swelling capabilities of our nanogels, combined with the maintained sink conditions, are paramount in governing the observed drug release characteristics of our formulations, offering valuable insights into their potential behavior within the gastrointestinal tract.

High variability observed in Figure 5 and Figure 6 could be attributed to several factors. One key reason could be the variability in curcumin dissolution behavior. Curcumin has poor aqueous solubility, which could lead to inconsistent dissolution across replicates. Furthermore, photodegradation and thermal degradation may also influence the dissolution profile. The observed improvements in dissolution and stability under simulated gastric and intestinal conditions are prerequisites for successful oral bioavailability, and future in vitro cell studies (e.g., Caco-2 permeability) and in vivo studies are needed to further investigate enhanced oral drug delivery.

#### 2.3.2. Morphology

**Figure 7:**
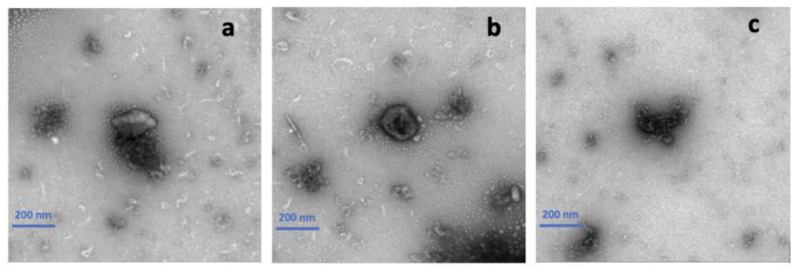
Transmission electron microscopy (TEM) images of (**a**) F10 (HA-g-pNIPAM physically mixed with curcumin), (**b**) F10 Encap (curcumin encapsulated within HA-g-pNIPAM), and (**c**) F11 (curcumin granules without HA-g-pNIPAM).

TEM was applied to verify the size and shape of the curcumin encapsulated in HA-g-pNIPAM. Also, it helped confirm that HA-g-pNIPAM remained after the solidification. The previous study showed that the particle size of curcumin loaded in the nanogel was around 100–300 nm [9]. From Figure 7a, we could observe the HA-g-pNIPAM nanogel and curcumin separately. As Figure 7b suggested, curcumin was incorporated in HA-g-pNIPAM which had a spherical shape and size of around 100–300 nm. From Figure 7c, F11 showed curcumin particles which could be used to interpret that curcumin was encapsulated in the nanogel.

#### 2.3.3. Particle Size of Nanoparticles

Particle size was measured to observe the size of HA-g-pNIPAM in solution (See Table 2). The findings showed that the particle size of both F10 and F10 Encap granules was sensitive to temperature, with the Z-average increasing as the temperature went up from 25 °C to 37 °C. Luckanagul et al. reported TEM analysis to confirm that the curcumin loaded in the nanogel exhibited a spherical shape with a particle size ranging between 100 and 300 nm [9]. Similarly, dynamic light scattering (DLS) data further validated that the size of curcumin loaded in the nanogel was within the same range of 100–300 nm [9]. Additionally, the PDI values for these formulations indicated that the incorporation of curcumin into HA-g-pNIPAM led to a more varied size distribution than what was seen with the F10 granules. This could be due to the additional complexity introduced by the encapsulation process, which might lead to a more heterogeneous population of particles. The enlargement of particle size and the wider size distribution observed in F10 Encap might have significant effects on the drug release characteristics and the bioavailability of curcumin. The temperature-dependent change in particle size and the broader size distribution observed in F10 Encap granules highlighted the importance of considering the physicochemical properties of drug delivery systems when designing formulations for therapeutic applications in the future. The data was reported in Z-average, and the particle size distribution spectra of nanoparticles are provided in Supplementary Figure S1.

**Figure 8:**
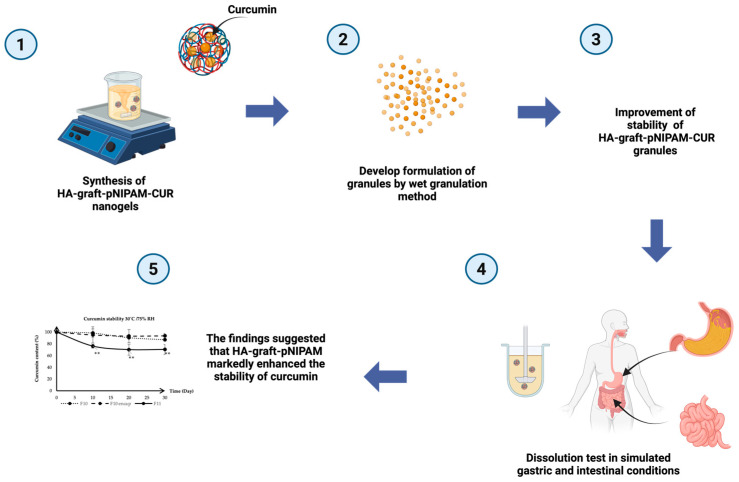
Schematic diagram of this research. ** indicates statistical significance at *p* < 0.05 (*n* = 3).

The schematic diagram (see Figure 8) provided a comprehensive visual summary of our research methodology, the development of the curcumin-loaded HA-g-pNIPAM nanogel granules, and the key findings related to enhanced stability and dissolution for oral drug delivery. The process started from the (1) synthesis of curcumin-loaded HA-g-pNIPAM nanogels, forming the foundational drug delivery system. These nanogels were then (2) successfully incorporated into granules via a wet granulation method. This formulation approach led to a significant (3) improvement in the stability of the HA-g-pNIPAM-CUR granules. The in vitro performance of these granules was rigorously assessed through (4) dissolution tests conducted under simulated gastric and intestinal conditions. Ultimately, these evaluations consistently demonstrated that (5) the HA-g-pNIPAM formulation markedly enhanced the stability of curcumin granules, thereby achieving the objective of the study.

## 3. Conclusions

This study explored curcumin formulations, with a particular focus on enhancing its dissolution profile and stability. The research was conducted through a series of experiments that included the formulation of curcumin with HA-g-pNIPAM, PVP K-30, L-HPC, and mannitol, and the evaluation of their physicochemical properties and in vitro dissolution profiles. The F10 Encap formulation, which incorporated curcumin encapsulated within HA-g-pNIPAM, demonstrated better stability at day 30 compared to the non-encapsulated F11 formulation. Scanning electron microscopy (SEM) imaging provided insights into the granule morphology, which was further correlated with the dissolution behavior observed. Dissolution studies under simulated gastric and intestinal conditions revealed distinct release profiles for each formulation. F10 Encap exhibited a more controlled and sustained release of curcumin, which is advantageous for therapeutic applications. The encapsulation process appeared to protect curcumin from premature release in the stomach’s acidic environment and promote its release in the more alkaline intestinal environment. The findings suggested that HA-g-pNIPAM markedly enhanced the stability of curcumin, minimizing its degradation and preserving its therapeutic efficacy for a longer duration. Further research is needed to assess the colloidal stability offered by the polymer matrix and to determine the influence of nanogels on drug loading capacity. Although this study achieved considerable progress in improving the solubility and stability of curcumin, there remained several aspects that warrant further exploration. These include the exploration of additional excipients that could further improve curcumin’s bioavailability, the conduct of in vivo studies to assess the pharmacokinetics and pharmacodynamics of the optimized formulations, and the investigation of curcumin’s synergistic effects with other therapeutic agents.

## 4. Materials and Methods

### 4.1. Preparation of the HA-g-pNIPAM Nanogels

Hyaluronic acid or HA was purchased from Liuzhou Shengqing Biotech Co., Ltd. (Guangxi, China). The molecular weight is 45–65 kDa. Poly(N-isopropylacrylamide) or pNIPAM was purchased from Sigma-Aldrich (MO, USA). Its molecular weight was 5500 Da. HA-g-pNIPAM polymer was synthesized via an EDC/NHS coupling reaction. Sodium hyaluronate was dissolved in ultrapure water. The reaction proceeded at room temperature for 48 h, and the product was purified by 3-day dialysis before freeze-drying. HA-g-pNIPAM nanogels were subsequently prepared by dissolving the polymer in ultrapure water (0.5% *w*/*v*) and sonication for 30 min, followed by overnight settling at 4 °C. All preparation process was described in a previous study [8].

### 4.2. Preparation of Cur-HA-g-pNIPAM

The curcumin analytical standard was purchased from Sigma-Aldrich (MO, USA) and has a molecular weight of 368.38 g/mol, presenting as a dark orange powder. Curcumin extract was procured from Naturalin Co., Ltd. (Hunan, China) and was derived from 100% natural dried *Curcuma Longa* roots, containing no less than 95% curcuminoids. Ethanol–water was used as the extraction solvent. The extract was characterized as a fine orange-yellow powder. Following the method was also outlined in the previous study [8].

### 4.3. Preparation of Granules by the Wet Granulation Method

Polyvinylpyrrolidone K-30 (PVP K-30) purchased from BASF Pharma (Ludwigshafen, Germany) was used as a binder and dispersing agent to facilitate the formation and stability of granules. Mannitol purchased from Sigma-Aldrich (MO, USA) was used as a filler to improve the flow properties and compressibility of the granules. Low-substituted hydroxypropyl cellulose (L-HPC) purchased from Shin-Etsu Chemical (Tokyo, Japan) primarily functioned as a disintegrating and binding agent, contributing to increased tablet hardness and surface smoothness while facilitating the disintegration process. Curcuminoid extract served as the main active ingredient, providing therapeutic effects.

We explored various ratios of curcuminoid extracts to identify the optimal concentration that balanced the solubility, stability, and bioavailability of the final formulations and optimized the proportion of other excipients in the formulations (unpublished data). After extensive experimentation, we determined that a ratio of 50.5 g of curcuminoid extract per 100 g of total formulation components yielded the best overall performance for our goal of developing a tablet formulation in the future. Three distinct formulations were prepared (see Table 3): (1) F10 (HA-g-pNIPAM physically mixed with curcumin), (2) F10 Encap (curcumin encapsulated within HA-g-pNIPAM), and (3) F11 (Curcumin granules without HA-g-pNIPAM).

For F10 (HA-g-pNIPAM physically mixed with curcumin), preparation of curcumin granules via the wet granulation method begins by accurately weighing PVP K-30, mannitol, L-HPC, and curcuminoid extract (see Table 3). Next, blend these dry ingredients evenly to avoid lumps with a cubic mixer for 25 min. Then, HA-g-pNIPAM solution with a concentration of 0.5% W/W will be added onto the mixture of dry powder, to serve as a moistening agent. Gradually incorporate this solution into the dry mix while stirring to create a wet mass. Pass this mass through a sieve to granulate with sieve no.16 and dry the granules in an oven at 40 °C for 30 min. After heating, the granules will be sieved with no.18 sieve. For F10 Encap (curcumin encapsulated within HA-g-pNIPAM), the weighting and mixing protocol are the same as F10 (see Table 3). Apart from the previous protocol, curcuminoid extract will be prior encapsulated in HA-graft-pNIPAM before being added to the formulation. The method to prepare curcumin loaded in HA-g-pNIPAM was mentioned in Section 4.2 Preparation of curcumin loaded in HA-g-pNIPAM (CUR-HA-g-pNIPAM). For F11 (curcumin granules without HA-g-pNIPAM), the weighting and mixing protocol are the same as F10 (see Table 3). Besides the earlier procedure, no HA-g-pNIPAM will be added to the formulation. Ultra-pure water will be the wetting agent.

### 4.4. HPLC Analysis of Curcumin Content

The curcumin content was analyzed using high-performance liquid chromatography (HPLC) with an Agilent 1260 Infinity II system (Agilent Technologies Inc., Santa Clara, CA, USA). The system comprised a quaternary pump (G7111A), a UV-Vis detector (G7115A), and an autosampler (G7129A), controlled by ChemStation software (version E.02.02). The content determination followed the United States Pharmacopeia (USP) methodology [11]. The HPLC analysis employed isocratic elution with a mobile phase consisting of 60% 0.1% *w*/*v* citric acid and 40% tetrahydrofuran (THF) purchased from Sigma-Aldrich (MO, USA) pat a flow rate of 1 mL/min. The column temperature was maintained at 40 °C, and detection was performed at 420 nm using a diode array detector. The sample injection volume was 20 µL, with a total run time of 20 min, and curcumin’s retention time was noted at 14 min.

Dried curcumin granules were dissolved in acetone and diluted with mobile phase before analysis. For drug content determination, 0.2 g of curcumin loaded in HA-g-pNIPAM granules and 0.2 g of curcumin standard were weighed and dissolved in the mobile phase and sonicated for 15 min. The solutions were then diluted to 10 mL with the mobile phase. After that, 500 µL of the solution was taken and made up to 10 mL with the mobile phase. The solution was filtered by a 0.22 µm filter and measured by reverse-phase HPLC (RP-HPLC). The linearity was *y* = 0.0907*x* + 168.6 where *y* represented the peak area (detector response), which was proportional to the amount of curcumin, and *x* represented the concentration of curcumin in ng/mL. Slope (0.0907) indicated the sensitivity of the HPLC method, and the intercept (168.6) reflected the background signal or baseline noise when no curcumin was present.

### 4.5. Stability of Curcumin in the Formulations

The three formulations were stored under conditions of 30 °C and 75% relative humidity, as well as 40 °C and 75% relative humidity, for a period of 30 days. At each time point, the granules will be analyzed by RP-HPLC method mentioned above.

### 4.6. Characterization of Dried Curcumin Granules

- Moisture content: A 2 g sample of the granules was placed on an aluminum tray, and the moisture content was measured with a moisture analyzer. The measurement was recorded once the reading stabilized, indicating the percentage of residual moisture.
- Bulk Density: The granules were measured by weight and then carefully poured into the upper funnel until they spilled over into the collection cup. The top of the collection tube will be evened out with a spatula to ensure it is filled. The bulk density will be determined by employing the following Formula (1):

(1) $$Bulk\;density=\frac{mass\;of\;the\;granules}{the\;volume\;initially\;held\;by\;the\;granules}$$

- Tapped density: Following the bulk density test, the granules were subjected to 500 tapping cycles. The volume after tapping was recorded and the tapped density was computed using Equation (2) as follows:

(2) $$Tapped\;density=\frac{mass\;of\;the\;granules}{the\;volume\;of\;granules\;following\;500\;tappings}$$

- Carr’s Index: Carr’s Index was calculated using the bulk and tapped density data, which indicated the granules’ compressibility. If the Carr’s Index is less than or equal to 15%, the granules are predicted to have good compressibility [13]. The Carr’s Index was determined using Formula (3):

(3) $$Carr\prime s\;Index=\frac{tapped\;density-bulk\;density}{tapped\;density}$$

- Angle of repose: The procedure outlined in the US Pharmacopeia General Chapter 1174 [13] was adhered to. The position of the funnel was fixed at 4 cm above the base plate. The orifice of the funnel is 10 mm. The 50 g samples were gently poured into the funnel, and the granules were stirred if required. The samples were gently poured into the funnel, and the granules were stirred if required. After the granules were accumulated into a conical pile on the platform, the height of the powder from the platform to the highest point of the cone (in millimeters) was measured. The calculation was based on the following Equation (4):

(4) $$Tan\left(\alpha \right)=\frac{height\;of\;the\;pile\;powder\;(mm)}{0.5*diameter\;(mm)}$$

- Morphology was analyzed by scanning electron microscope (SEM) by using model JSM-IT800 (JEOL, Japan).
- Particle size distribution was measured by dry sieve analysis mentioned in the US Pharmacopeia [25].

### 4.7. Characterization of Curcumin Reconstituted Granules

- Dissolution test: in vitro dissolution tests were conducted on formulations F10, F10 Encap, and F11 to assess the solubility and stability of curcumin within each preparation. A dissolution study was performed based on the previous method [3,4] using a type II dissolution apparatus. For the dissolution tests, 200 mg portions of each formulation, which included 100 mg of curcumin, were introduced into dissolution vessels filled with 500 mL of simulated intestinal fluid and simulated gastric conditions. Gastric fluid was made from dissolving 2.0 g of sodium chloride and 3.2 g of pepsin USP grade in 500 mL of water. Then 7.0 mL of hydrochloric acid was added and diluted to 1000 mL with water. The pH was adjusted to 1.2. For intestinal fluid, 6.8 g of potassium phosphate (monobasic) was added to 250 mL of water. After that, 190 mL of sodium hydroxide solution (0.2 M) and 400 mL of water were mixed in the same container. 10.0 g of pancreatin USP grade was added, and the pH of the resulting solution was adjusted to 7.5 ± 0.1 with sodium hydroxide solution (0.2 M). The suspension was diluted with water to make 1000 mL. These conditions were maintained at a temperature of 37 ± 0.1 °C, and the vessels were agitated at a speed of 100 revolutions per minute (rpm). Samples were taken at specified time intervals, and an equal volume of fresh dissolution medium was added to each vessel to maintain the initial volume. A 10 mL aliquot of the sample was removed and subjected to lyophilization to convert it into a solid form. Appropriate dilution was performed, and drug content was analyzed by RP-HPLC (Agilent 1260 Infinity, USA) using DAD detector [11].
- Morphology: The morphology of the curcumin encapsulated with HA-g-pNIPAM was evaluated by transmission electron microscopy (TEM). The granules were dissolved and diluted with pure water: ethanol (1:1). The samples were negatively stained with 1% uranyl acetate on a copper grid for 3 min and dried at room temperature for 3 h under dust-free conditions. Then, the dried samples were examined under a transmission electron microscope, Thermo Scientific Talos F200X G2 TEM, and recorded as magnified images to observe the surface morphology and the size of the nanoparticles.
- The particle size was determined using Dynamic Light Scattering (DLS) with a Zetasizer Nano ZS from Malvern Instruments, UK.

### 4.8. Materials

Hyaluronic acid was purchased from Liuzhou Shengqing Biotech Co., Ltd. (Guangxi, China). Poly(N-isopropylacrylamide), curcumin analytical standard, mannitol, pepsin USP grade, and pancreatin USP grade were purchased from Sigma-Aldrich (MO, USA). Polyvinylpyrrolidone K-30 (PVP K-30) was purchased from BASF Pharma, Ludwigshafen, Germany. Low-substituted hydroxypropyl cellulose (L-HPC) was purchased from Shin-Etsu Chemical, Tokyo, Japan. Citric acid and tetrahydrofuran (THF) were purchased from Sigma-Aldrich (MO, USA).

### 4.9. Statistical Methods

All data were collected in triplicate and expressed as the mean ± standard deviation (S.D.). Statistical analysis was conducted using SPSS 29.01.0 software, with one-way ANOVA used to evaluate the statistical difference among groups. A *p*-value of <0.05 was considered statistically significant.

## Figures and Tables

**Table 1:** Physicochemical properties of dried curcumin granules.

Characteristics of the Granules	Moisture Content (%w/w)	Bulk Density (g/mL)	Tapped Density (g/mL)	Carr’s Index	Type of Flow (Carr’s Index)	Angle of Repose	Type of Flow (Angle of Repose)
F10 (HA-g-pNIPAM physically mixed with curcumin)	2.80 ± 0.21	0.45 ± 0.08	0.55 ± 0.11	16.67%	Fair	9.5°	Excellent
F10 Encap (curcumin encapsulated within HA-g-pNIPAM)	3.07 ± 0.20	0.46 ± 0.06	0.55 ± 0.06	16.67%	Fair	10°	Excellent
F11 (curcumin granules without HA-g-pNIPAM)	3.03 ± 0.15	0.47 ± 0.09	0.56 ± 0.14	15%	Good	10.33°	Excellent

Note: Data are presented as mean ± standard deviation (S.D.) (*n* = 3). Statistical analysis was performed using one-way ANOVA; no significant differences were observed among formulations (*p* < 0.05).

**Table 2:** Particle size in nanometers and the polydispersity index (PDI).

Sample	Z-Average (nm)	PDI
F10 at 25 °C	163.60 ± 9.35	0.41 ± 0.01
F10 Encap at 25 °C	213.50 ± 1.59	0.54 ± 0.03
F10 at 37 °C	204.17 ± 2.08	0.37 ± 0.04
F10 Encap at 37 °C	236.23 ± 15.69	0.55 ± 0.06

Note: Data are presented as mean ± standard deviation (S.D.) (*n* = 3). F10 stands for HA-g-pNIPAM physically mixed with curcumin, and F10 Encap symbolizes curcumin encapsulated within HA-g-pNIPAM.

**Table 3:** Formulation of granules.

Formulation and Ingredients	F10 (HA-g-pNIPAM Physically Mixed with Curcumin) (g, wt%)	F10 (HA-g-pNIPAM Physically Mixed with Curcumin) (g, wt%)	F10 (HA-g-pNIPAM Physically Mixed with Curcumin) (g, wt%)
PVP K-30	5	5	5
Mannitol	19.5	19.5	19.5
L-HPC	5	5	5
Curcuminoid extracts	50.5	50.5 (8.736 mg encapsulated curcumin)	50.5
HA-g-pNIPAM solution	20	20	0
Ultra-pure water	0	0	20
Total Weight (g)	100	100	100

## Data Availability

No data was used for the research described in the article.

## Supplementary Materials

The following supporting information can be downloaded at: https://www.mdpi.com/article/10.3390/gels11080585/s1, Table S1. Carr’s Index and flow character; Table S2. Angle of repose and flow character; Figure S1. Spectra of particle size obtained from Zetasizer.
