# Supplementary material for: Development of a Curcumin-Loaded Hyaluronic Acid Nanogel Formulation Using Wet Granulation Method for Enhanced Dissolution and Stability
Source: Gels. 2025 Jul 29;11(8):585. doi: 10.3390/gels11080585 (PMC12385313; doi:10.3390/gels11080585)

gels

MDPI

Supplementary

Development of a Curcumin-Loaded Nanogel Containing Hyaluronic Acid Formulation for Oral Drug Delivery Using Wet Granulation Method

Natkhanang Boonpetcharat [1], May Thu Thu Kyaw [1], Veerakiet Boonkanokwong [1] and Jittima Amie Luckanagul [1,2,*]

Table S1. Carr's Index and flow character

Carr's Index (%)	Flow Character
≤10	Excellent
11-15	Good
16-20	Fair
21-25	Passable
26-31	Poor
32-37	Very poor
>38	Very, very poor

Table S2. Angle of repose and flow character

Angle of repose (degrees)	Flow Character
25-30	Excellent
31-35	Good
36-40	Fair – aid not needed
41-45	Passable – may hang up
46-55	Poor – must agitate, vibrate

56-65	Very poor
>66	Very, very poor

Table S1 and Table S2 followed USP-NF <1174> Powder Flow[1]. When the angle of repose exceeds 50°, the flow is rarely acceptable for manufacturing purposes.

[1]United States Pharmacopeia. <1174> Powder flow. 2024, *No.* 46(3), doi:https://doi.org/10.31003/USPNF_M99885_02_01.

Figure S1. Spectra of particle size obtained from Zetasizer

(a) F10 at 25°C

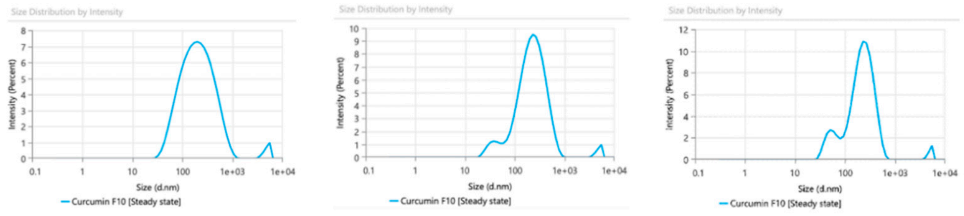

(b) F10 at 37°C

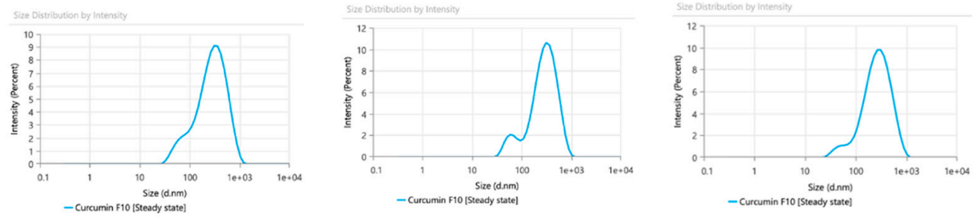

(c) F10 Encap at 25°C

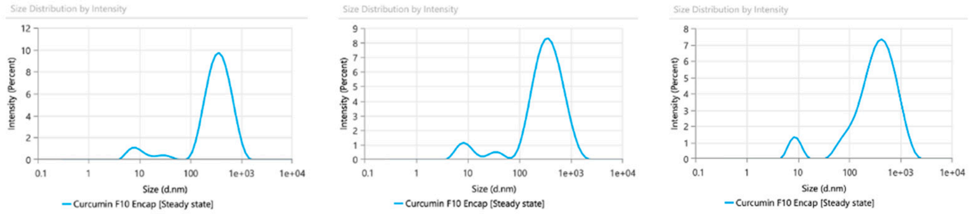

(d) F10 Encap at 37°C

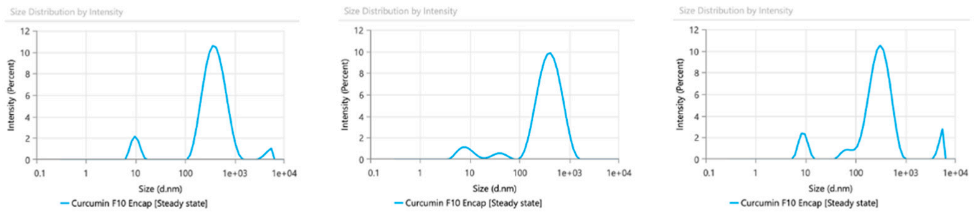

Supplement: Supplementary file 1 [file gels-11-00585-s001.zip › gels-3717752-supplementary.pdf]
